# Esophageal Cancer in Kazakhstan: Multi-omic Research Challenges

**DOI:** 10.5195/cajgh.2014.170

**Published:** 2014-12-12

**Authors:** Saule Rakhimova, Ainur Akilzhanova, Yurii Zhukov, Marat Omarov, Zhaxybay Zhumadilov

**Affiliations:** 1 Center for Life Sciences, Nazarbayev University, Astana, Kazakhstan; 2 Oncology Center, Astana, Kazakhstan

**Keywords:** esophageal cancer, Kazakhstan, RNA sequence, squamous cell carcinoma

## Abstract

**Introduction:**

Esophageal cancer (EC) is the sixth most common cancer in Kazakhstan, fifth leading cause of mortality among men, and ninth leading cause of mortality among women. Advances in high-throughput sequencing over the last decade have made mapping the whole genetic variation in genome-wide scale possible. Transcriptome sequencing has become a powerful method for detecting driver mutations in cancer, since somatic point mutations as well as aberrant RNA variants, such as fusion genes and alternative splicing, can be identified. The aim of the study was to identify the genetic basis of EC by performing whole transcriptome sequencing (RNA-Seq) study in Kazakhstani patients.

**Materials and methods:**

We included patients with EC who had been admitted to the oncology center in Astana, Kazakhstan during the 2013–2014 year period. A pair of fresh frozen EC, its adjacent normal tissue specimen, and venous blood were obtained. So far, five pairs of EC samples were subjected to RNA-seq. Total RNA was isolated, and its quality was assessed using Agilent Bioanalyzer. The cDNA library was prepared following the standard mRNA protocol by Illumina and sequenced using Illumina HiSeq2000. Bionformatic analysis is ongoing.

**Results:**

During 2013, a total of 74 patients with EC were hospitalized in the oncology center, Astana, Kazakhstan. Radical and palliative surgery was performed on 39 and 34 patients, respectively, and 1 patient refused surgery treatment. The median age of the patients was 66 years (range 49–86 years). 88.4% of the patients were diagnosed with advanced stages T3–T4, and 74.5% from them has dysphagia III–IV levels. 83% of the cases were squamous cell carcinoma (ESCC). The major localizations for this type of cancer were the middle section (58.2%), lower section (37.2%), and upper section (4.6%) of the cases.

**Conclusion:**

ESCC is the most common histologic subtype of esophageal cancer in our patients and is characterized by a poor prognosis. Most patients were diagnosed with late stages T3–T4. Using high throughput sequencing approach, we could potentially identify a higher number of crucial molecular pathways involved in esophageal carcinogenesis that could facilitate the development of new diagnostic and treatment strategies. The early detection of EC gives hope of a long-term survival for patients.

